# A Risk-Factor Model for Antineoplastic Drug-Induced Serious Adverse Events in Cancer Inpatients: A Retrospective Study Based on the Global Trigger Tool and Machine Learning

**DOI:** 10.3389/fphar.2022.896104

**Published:** 2022-06-29

**Authors:** Ni Zhang, Ling-Yun Pan, Wan-Yi Chen, Huan-Huan Ji, Gui-Qin Peng, Zong-Wei Tang, Hui-Lai Wang, Yun-Tao Jia, Jun Gong

**Affiliations:** ^1^ National Clinical Research Center for Child Health and Disorders, Chongqing Key Laboratory of Pediatrics, Department of Pharmacy, Children’s Hospital of Chongqing Medical University, Ministry of Education Key Laboratory of Child Development and Disorders, Chongqing, China; ^2^ School of Pharmacy, Chongqing Medical University, Chongqing, China; ^3^ Department of Pharmacy, Chongqing University Cancer Hospital, Chongqing, China; ^4^ Department of Information Center, The University Town Hospital of Chongqing Medical University, Chongqing, China

**Keywords:** antineoplastic drugs, machine learning, serious adverse events, Global Trigger Tool, prediction

## Abstract

The objective of this study was to apply a machine learning method to evaluate the risk factors associated with serious adverse events (SAEs) and predict the occurrence of SAEs in cancer inpatients using antineoplastic drugs. A retrospective review of the medical records of 499 patients diagnosed with cancer admitted between January 1 and December 31, 2017, was performed. First, the Global Trigger Tool (GTT) was used to actively monitor adverse drug events (ADEs) and SAEs caused by antineoplastic drugs and take the number of positive triggers as an intermediate variable. Subsequently, risk factors with statistical significance were selected by univariate analysis and least absolute shrinkage and selection operator (LASSO) analysis. Finally, using the risk factors after the LASSO analysis as covariates, a nomogram based on a logistic model, extreme gradient boosting (XGBoost), categorical boosting (CatBoost), adaptive boosting (AdaBoost), light-gradient-boosting machine (LightGBM), random forest (RF), gradient-boosting decision tree (GBDT), decision tree (DT), and ensemble model based on seven algorithms were used to establish the prediction models. A series of indicators such as the area under the ROC curve (AUROC) and the area under the PR curve (AUPR) was used to evaluate the model performance. A total of 94 SAE patients were identified in our samples. Risk factors of SAEs were the number of triggers, length of stay, age, number of combined drugs, ADEs occurred in previous chemotherapy, and sex. In the test cohort, a nomogram based on the logistic model owns the AUROC of 0.799 and owns the AUPR of 0.527. The GBDT has the best predicting abilities (AUROC = 0.832 and AUPR = 0.557) among the eight machine learning models and was better than the nomogram and was chosen to establish the prediction webpage. This study provides a novel method to accurately predict SAE occurrence in cancer inpatients.

## 1 Introduction

Cancer is a constant challenge for public health in the world. It has become the second leading cause of death after cardiovascular disease which seriously threatens human health. The statistical report announced by the International Agency for Research on Cancer (IARC) in 2020 predicts that the global cancer burden is expected to reach 29 million new cancer cases per year until 2040, an increase of 62% over the estimated 18.1 million cancers in 2018 (Wild Cp, 2020). As the most populous country in the world, China accounts for about 23% of new cancer cases and 30% of cancer deaths (Hyuna et al., 2021). A survey shows that the direct economic burden caused by cancer in China was $221.4 billion which accounted for 5.4% of the total health expenditure and 17.7% of the government’s public health expenditure in 2015 (Cai et al., 2017).

With the increasing incidence rate of cancer, the research on the methods of treating cancer has also been deepened. The increasing antineoplastic drugs such as molecular targeted therapy and immunotherapy have effectively controlled many cancers. However, the drug-induced safety problems cannot be ignored, which not only affect the treatment of patients but also some patients interrupt treatment or even die because of serious adverse events (SAEs) caused by antineoplastic drugs (Zhiwei et al., 2019). Compared with clinical trials, patients who receive chemotherapy have a higher frequency of SAEs in clinical practice, which has been reported in the systematic evaluation of lung cancer treatment (Prince et al., 2015). A retrospective study from Japan that investigated the types and frequencies of SAEs after oral antineoplastic drugs in outpatients has found that SAEs usually occurred early after the beginning of the treatment (Kenji et al., 2021). SAEs led to deterioration in the quality of life, increased healthcare costs, and earlier morbidity and mortality (Bates et al., 1995). Hence, SAEs in cancer patients were considered an important event with high clinical value. Early identification and warning of individuals associated with SAEs are particularly important.

The Global Trigger Tool (GTT) was first proposed by the Institute for Healthcare Improvement (IHI) in 2003; it is a commonly used method for identifying potential adverse drug events (ADEs) among cancer inpatients (Lipitz-Snyderman et al., 2017). Anne et al. (2020) described the ADEs of cancer patients with the GTT, in which the positive predictive value (PPV) was 42%. Christin et al. (2017) used the GTT to investigate whether hospitalized cancer inpatients are at higher risk of ADEs than a general hospital population, and it has been found that higher age, longer length of stay, and surgical treatment were the risk factors of ADEs in cancer inpatients compared with other patients. Although certain studies have reported a variety of predictive factors for ADEs, such as patient illness severity, patient increased age (>65 years), receiving more than five drugs, and length of hospital stay, the findings are partly contradictory (Simon et al., 2011; Paul et al., 2011; Chuenjid et al., 2013; Qiaozhi et al., 2020). The GTT has certain capabilities in detecting ADEs, but some studies have shown that the GTT is not specific enough in studying the harm to cancer patients, and the PPV of the GTT is generally low, which varies greatly between different populations and medical centers (Otto et al., 2013).

Machine learning is a new artificial intelligence discipline, which has been widely used to assist doctors to make an objective judgment (de Mattos et al., 2022; Höppner, 2020). In this study, the GTT was first used to actively monitor the occurrence of ADEs and SAEs caused by antineoplastic drugs. Then, the machine learning method was used to explore the relevant risk factors of SAEs caused by antineoplastic drugs and construct predictive models, to make up for the poor performance of the GTT. Our study tries to establish a machine learning model to quantitatively predict the probability and degree of SAEs of antineoplastic drugs, to provide a risk prediction tool for clinical work and take effective measures.

## 2 Methods

### 2.1 Study Participants

A retrospective medical record’s review was performed for a random sample of 600 inpatients (50 per month) in Chongqing Cancer Hospital discharged from January 1 2017 to December 31, 2017. The inclusion criteria were patients diagnosed with cancer, whose length of stay >2 days and ≤30 days, and antineoplastic drugs used during hospitalization. The exclusion criteria were as follows: patients who had no antineoplastic drug exposure and had used traditional Chinese medicine to treat cancer.

This study is a retrospective study and the patients’ informed consent is not required. The protocol of this study has been approved by the Ethics Committee of Chongqing University Cancer Hospital (CZLS2022008-A) and the Ethics Committee of Chongqing Medical University.

### 2.2 Positive Cases

First, the GTT method was used to detect the occurrence of ADEs. Subsequently, two pharmacists were assigned to examine the data and determine the occurrence of ADEs. If there were disagreements, the final decision was made by a senior pharmacist. Finally, SAE patients were selected from all ADE patients according to CTCAE 5.0, and events with grades 3–5 were defined as SAEs (National Institutes Of Health and National Cancer Institute, 2017).

### 2.3 Candidate Predictors

The SAE risk factors were screened from multiple patient characteristics according to the results of previous research (Simon et al., 2011; Paul et al., 2011; Chuenjid et al., 2013; Qiaozhi et al., 2020). To be specific, we included the patients’ demographic information (such as sex, age, and weight), disease situation (such as cancer types and cancer stage), treatment information (such as number of antineoplastic drugs and number of combined drugs), and the number of GTT triggers. The occurrence of SAEs was used as the target variable to analyze which characteristic had a remarkable influence on it.

### 2.4 Statistical Analysis

The whole dataset was divided into training and test cohorts at the ratio of 8:2 according to a random number table. The training cohorts were used to select risk factors and establish the model, and the test cohorts were used to verify the performance of the model. All statistical computing was conducted in R for Windows (version 4.0.5, https://www.r-project.org/) and SPSS 25.0 (IBM Corporation, Armonk, NY, USA). *p* < 0.05 was considered to be statistically significant.

Data were presented as count with percentage for categorical variables, median with interquartile range, or mean with standard deviation for continuous variables. The Mann–Whitney U-test or T-test was performed for the continuous variables, and the Chi-square test for categorical variables. Least absolute shrinkage and selection operator (LASSO) analysis carried out used to explore the interaction of variables screened by the univariate analysis on the occurrence of SAEs. Subsequently, using the variables after the LASSO analysis as covariates, the nomogram based on the logistic model, extreme gradient boosting (XGBoost), categorical boosting (CatBoost), adaptive boosting (AdaBoost), light gradient boosting machine (LightGBM), random forest (RF), gradient boosting decision tree (GBDT), decision tree (DT) algorithms, and ensemble model based on seven machine learning algorithms were used to establish prediction models. Precision, recall, F1, sensitivity (SEN), specificity (SPE), area under the PR curve (AUPR), and area under the ROC curve (AUROC) were intended to determine the predictive ability. The evaluation indicator formulas were shown in our previous research (Ze et al., 2021). At the same time, we also performed a logistic analysis on the results of the univariate analysis and the established nomogram, compared with the results of the machine learning model. Ultimately, the algorithm with the best performance was selected to establish the model to predict the occurrence of SAEs.

## 3 Results

### 3.1 Study Population

**FIGURE 1:**
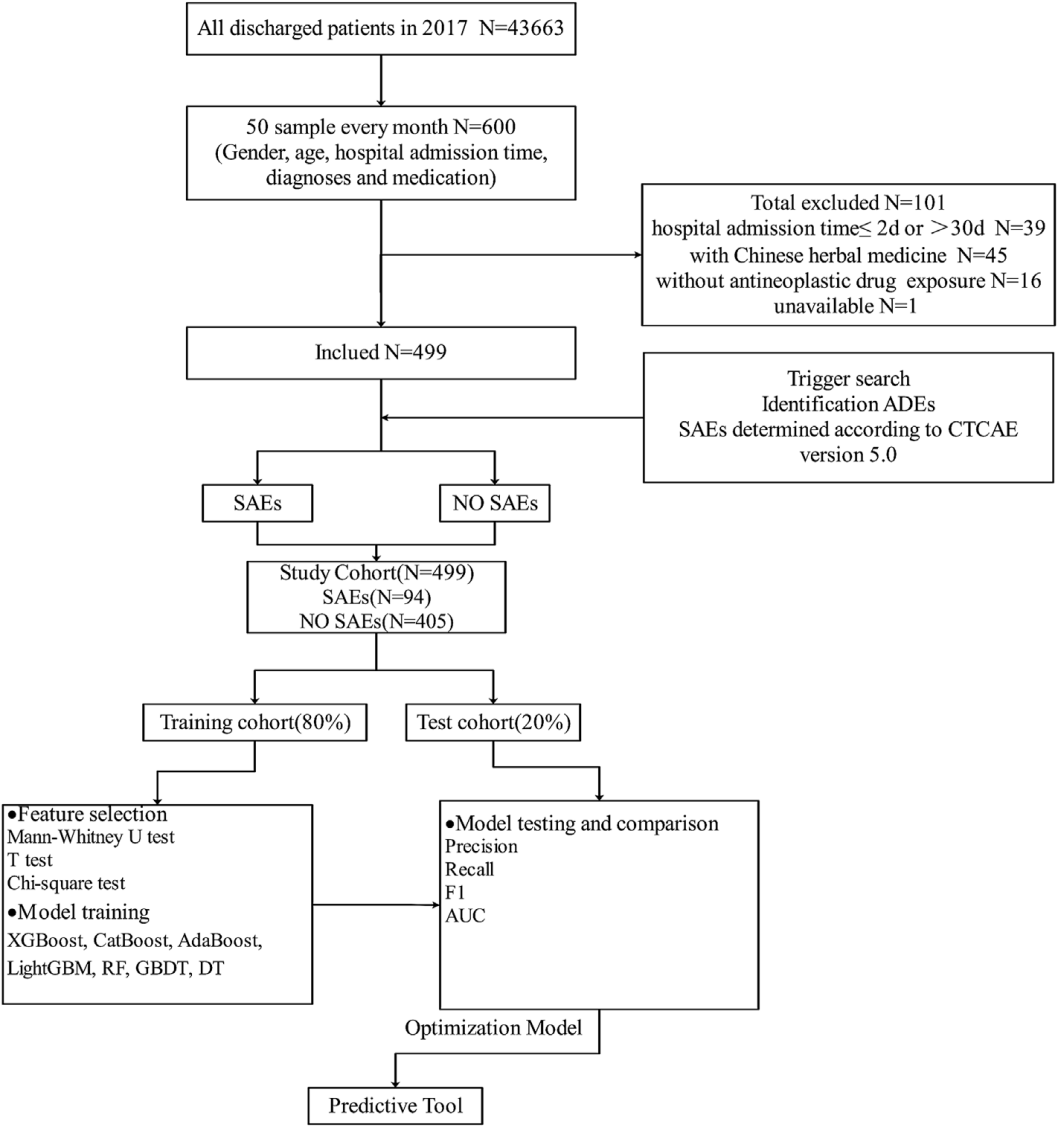
Overview of the study design and model development.

The hospital had 43,663 medical records from January–December 2017. According to the inclusion and exclusion criteria, a total of 499 patients (cases) were selected in this study. The specific screening process and study protocol are shown in Figure 1.

In the process of SAE identification, we established 33 kinds of triggers, among which 30 triggers were positive (90.91%) in our study. A total of 620 ADEs were identified from the 30 triggers. Among the 499 cases, 75.55% of patients had at least one ADE, and a total of 104 SAEs in 94 patients were recorded. The number of positive triggers, ADEs, SAEs, and trigger’s PPV are displayed in Table 1.

**TABLE 1:** Trigger items and their PPV.

No.	Trigger	ADEs	Positive trigger (n)	ADEs (n)	SAEs (n)	PPV (%)
Laboratory						
L1	Hb < 100 g L^−1^	Anemia	94	73	19	77.66
L2	Platelets count <100*10^9^ L^−1^	Thrombocytopenia	70	49	4	70
L3	Neutrophils <1.5*10^9^ L^−1^	Neutropenia	76	60	24	78.95
L4	Leukocyte count <3*10^9^ L^−1^	Leukopenia	128	109	36	85.16
L5	AST or CB > 2ULN; AST, ALP, and TBI elevated at least one of them >2×baseline)	Drug-induced hepatotoxicity	23	6	3	26.09
L6	GFR <60 ml/min or 50% greater than baseline*	Drug-induced renal toxicity	2	1	0	50
L7	Blood pressure >140/90 mmHg	Hypertension	63	2	0	3.17
L8	Blood glucose >8.9 mmol L^−1^	Drug-induced hyperglycaemia	11	3	0	27.27
L9	Blood glucose <3 mmol L^−1^	Drug-induced hypoglycemia	4	0	0	0
L10	Serum kalium >5.5 mmol L^−1^	Hyperkalemia	0	0	0	—
L11	Serum kalium <3.0 mmol L^−1^	Hypopotassemia	60	39	6	65
L12	Serum calcium >2.8 mmol L^−1^	Hypercalcemia	0	0	0	—
L13	Serum calcium <2.0 mmol L^−1^	Hypocalcemia	34	17	4	50
L14	Thyroid-stimulating hormone>4.2 mIU·L^−1^	Hypothyroidism	5	2	0	40
L15	Thyroid-stimulating hormone <0.34 mIU·L^−1^	Hyperthyroidism	1	1	0	100
L16	Serum uric acid elevated (Female>360 mol L^−1^, male>420 μmol L^−1^)	Hyperuricemia	30	21	4	70
L17	Positive qualitative test of urinary protein positive or urinary protein excretion> 150 mg per 24 h	Proteinuria	3	0	0	0
L18	Troponin>0.64 ng mL^−1^	Myocardial infarction	1	1	1	100
L19	BNP >400 pg mL^−1^ or NT-prBNP>2000 pg mL^−1^	Cardiac failure	13	3	1	23.08
Symptom						
S1	Oral mucositis	Oral mucositis	7	4	1	57.14
S2	Fever (body temperature>38.2°C)	Fever	1	1	0	100
S3	Diarrhea	Diarrhea	6	4	0	66.67
S4	Nausea or vomiting	Nausea or vomiting	199	186	0	93.47
S5	Constipation	Constipation	21	15	0	71.43
S6	Desquamation; erythema; redness	Hand–foot syndrome	2	2	0	100
S7	Rash	Rash	2	2	0	100
S8	Paresthesia; neuropathy; pins and needles; pain in hands and feet	Peripheral neuritis	1	1	0	100
S9	Extravasation	Extravasation	0	0	0	—
Medication						
M1	Corticosteroid and antihistamines use	Allergy	130	4	0	3.08
M2	Antithrombotic use	Thromboembolism	60	10	1	16.67
M3	Leucovorin use	Methotrexate poisoning	6	0	0	0
Treatment						
T1	Unplanned emergency treatment, resuscitation, or transfer to ICU	Emergency treatment, resuscitation, or transfer to ICU due to ADEs	0	0	0	—
T2	Unplanned adjust therapeutic regimen	Adjust therapeutic regimen due to ADEs	17	4	0	23.53

BNP, brain natriuretic peptide; ICU, intensive care unit; TSH, thyroid-stimulating hormone; AST, aspartate amino transferase; ALP, alkaline phosphatase; ULN, upper limit of normal; ADEs, Adverse drug events; PPV, positive predictive value.

In the whole cohort, the average age of patients was 53.97 ± 11.91 years, ranging from 13–88 years, females accounted for 61.32% (306 cases) and males 38.68% (193 cases). The mean length of stay was 9.32 ± 5.07 days (3–30 days). The most common type of cancer was breast cancer (121 cases, 24.25%), followed by lung cancer (102 cases, 20.44%) and lymphoma (56 cases, 11.22%). The cancer stage was mainly concentrated in stage Ⅲ∼ Ⅳ (326 cases, 65.33%), and Karnofsky performance status (KPS) scores were more than 70 before chemotherapy (449 cases, 89.98%). The relationships of these factors with the occurrence of SAEs need further screening in the following sections. According to table 2, there were 27 kinds of suspected drugs leading to SAEs, and the number of medications was 683 times; plant origin and other derivatives account for the largest proportion of suspected drugs of SAEs (31.77%), followed by platinum metal (24.16%), alkylating agent (16.40%), antineoplastic antibiotics (15.08%), antimetabolic drugs (12.15%), and molecular targeted drugs (0.44%).

**TABLE 2:** Classification of drugs leading to the occurrence of SAEs.

Classification of drugs	Suspected drugs	Number of cases (n)	Percentage (%)	Group percentage (%)
Platinum metal	Oxaliplatin	25	3.66	24.16
	Cisplatin	60	8.78	
	Nedaplatin	57	8.35	
	Carboplatin	23	3.37	
Antimetabolic drugs	Capecitabine	24	3.51	12.15
	Gemcitabine	22	3.22	
	Tegafur	12	1.76	
	Fluorouracil	8	1.17	
	Methotrexate	6	0.88	
	Cytarabine	2	0.29	
	Pemetrexed	7	1.02	
	Fludarabine	2	0.29	
Antineoplastic antibiotics	Pirarubicin	48	7.03	15.08
	Epirubicin	41	6.00	
	Bleomycin	11	1.61	
	Dactinomycin	3	0.44	
Plant origin and other derivatives	Paclitaxel	92	13.47	31.77
	Docetaxel	33	4.83	
	Vindesin	44	6.44	
	Etoposide	35	5.12	
	Irinotecan	7	1.02	
	Vinorelbine	6	0.88	
Alkylating agent	Cyclophosphamide	100	14.64	16.40
	Dacarbazine	12	1.76	
Molecular targeted drugs	Rituximab	1	0.15	0.44
	Trastuzumab	1	0.15	
	Bevacizumab	1	0.15	

### 3.2 SAEs and Risk Factors

**FIGURE 2:**
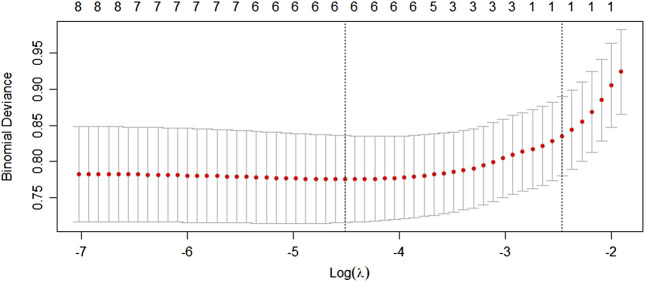
LASSO analysis after the univariate analysis.

According to Table 3, there is no significant difference between the training and test cohorts (*p* > 0.05), except that sex and radiation therapy have a slightly lower *p*-value (*p* < 0.05). Univariate analysis results indicated that eight variables were statistically significant between the SAE group and no SAE group in training cohorts, including sex, cancer type, ADEs occurred in previous chemotherapy, age, length of stay, number of previous chemotherapies, number of combined drugs, and number of triggers, while other eight variables were not statistically significant. We used the LASSO analysis to further screen the variables after the univariate analysis to avoid collinearity of variables and simplify the model variables. The result suggested that the log of the optimal value of lambda was 6 (Figure 2). Thus, six variables were selected as machine learning model predictors. They are sex, ADEs occurred in previous chemotherapy, age, length of stay, number of previous chemotherapies, and number of triggers.

**TABLE 3:** Characteristics of patients with and without SAEs.

Characteristic	Training cohort (N = 399)	Test cohort (N = 100)	*p*	Patients with no SAEs in the training cohort (N = 330)	Patients with SAEs in the training cohort (N = 69)	*p*
Sex (male)	164 (41%)	29 (29%)	0.026	143 (43%)	21 (30%)	0.048
Age (year)	53 (46, 63)	52 (48, 61)	0.787	53 (47, 63)	49 (42, 55)	0.001
Length of stay (days)	8.0 (6.0, 12.0)	8.0 (6.0, 10.2)	0.702	7.0 (6.0, 11.0)	10.0 (7.0, 15.0)	<0.001
Weight (kg)	58 (53, 63)	59 (51, 63)	0.740	58 (53, 63)	57 (52, 62)	0.800
Off-label drug use (yes)	104 (26%)	19 (19%)	0.143	86 (26%)	18 (26%)	0.996
Cancer type			0.508			0.007
Breast cancer	93 (23%)	28 (28%)		75 (23%)	18 (26%)	
Lung cancer	81 (20%)	21 (21%)		75 (23%)	6 (8.7%)	
Lymphoma	48 (12%)	8 (8%)		35 (11%)	13 (19%)	
Gastrointestinal	48 (12%)	10 (10%)		45 (14%)	3 (4.3%)	
Genital system	64 (16%)	21 (21%)		51 (15%)	13 (19%)	
Others	65 (16%)	12 (12%)		49 (15%)	16 (23%)	
Cancer stage			0.494			0.873
Ⅰ	43 (11%)	13 (13%)		37 (11%)	6 (8.7%)	
Ⅱ	92 (23%)	25 (25%)		74 (22%)	18 (26%)	
Ⅲ	120 (30%)	34 (34%)		100 (30%)	20 (29%)	
Ⅳ	144 (36%)	28 (28%)		119 (36%)	25 (36%)	
Operation (yes)	115 (29%)	27 (27%)	0.718	98 (30%)	17 (25%)	0.399
Basic diseases (yes)	93 (23%)	22 (22%)	0.781	79 (24%)	14 (20%)	0.514
Radiation therapy (yes)	21 (5.3%)	15 (15%)	0.001	19 (5.8%)	2 (2.9%)	0.333
ADEs occurred in previous chemotherapy (yes)	119 (30%)	35 (35%)	0.316	90 (27%)	29 (42%)	0.015
Number of previous chemotherapies	2.0 (0.0, 4.0)	2.0 (0.0, 3.0)	0.369	2.00 (0.00, 4.00)	3.00 (1.00, 5.00)	0.004
KPS			0.200			0.251
60	3 (0.8%)	0 (0%)		3 (0.9%)	0 (0%)	
70	43 (11%)	4 (4.0%)		34 (10%)	9 (13%)	
80	132 (33.3%)	34 (34%)		115 (35.3%)	17 (25%)	
90	192 (48%)	50 (50%)		156 (47%)	36 (52%)	
100	29 (7.3%)	12 (12%)		22 (6.7%)	7 (10%)	
Number of antineoplastic drugs			0.892			0.160
1	50 (13%)	10 (10%)		42 (13%)	8 (12%)	
2	254 (64%)	69 (69%)		214 (65%)	40 (58%)	
3	53 (13%)	13 (13%)		44 (13%)	9 (13%)	
4	26 (6.5%)	4 (4.0%)		20 (6.1%)	6 (8.7%)	
5	15 (3.8%)	4 (4.0%)		9 (2.7%)	6 (8.7%)	
6	1 (0.3%)	0 (0%)		1 (0.3%)	0 (0%)	
Number of combined drugs	5.00 (4.00, 7.00)	5.00 (4.00, 7.00)	0.531	5.00 (4.00, 6.00)	6.00 (4.00, 8.00)	0.018
Number of triggers	2.00 (1.00,3.00)	2.00 (1.00,3.00)	0.187	1.00 (1.00,2.00)	3.00 (2.00,4.00)	<0.001

### 3.3 Logistic Model and Nomogram Establishment

**FIGURE 3:**
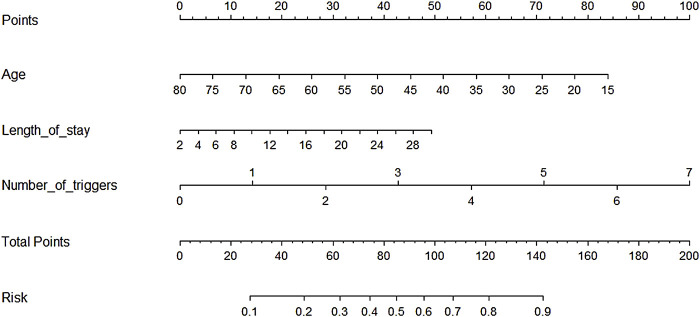
Nomogram based on the logistic model.

**FIGURE 4:**
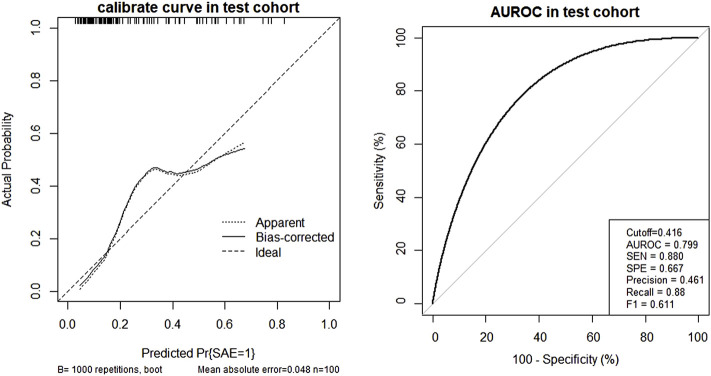
Nomogram calibration curve and AUROC in the test cohort.

To build a risk-factor model, the six variables which were statistically significant were used as input variables, and whether SAEs occurred after the use of antineoplastic drugs was regarded as the outcome event (yes = 1, no = 0) to establish the prediction model. The results of the stepwise forward logistic regression showed that age, length of stay, and number of triggers were screened and entered into the final model (Table 4). We have drawn a nomogram based on these three indicators (Figure 3), and added up the points of each indicator that could get the probability of SAEs occurrence. The test cohort was used to verify the performance of the nomogram. Among the test cohort, the Brier of the nomogram was 0.189, the AUPR was 0.527, and the AUROC was 0.779 (Figure 4), indicating that the model had a good performance.

**TABLE 4:** Logistic regression for SAEs.

Variables	B value	*p* value	OR	95% CI
Age	-0.033	0.004	0.967	0.945, 0.990
Length of stay	0.064	0.017	1.067	1.011, 1.125
Number of triggers	0.635	<0.001	1.886	1.531, 2.323

### 3.4 Machine Learning Model Establishment and Comparison

**FIGURE 5:**
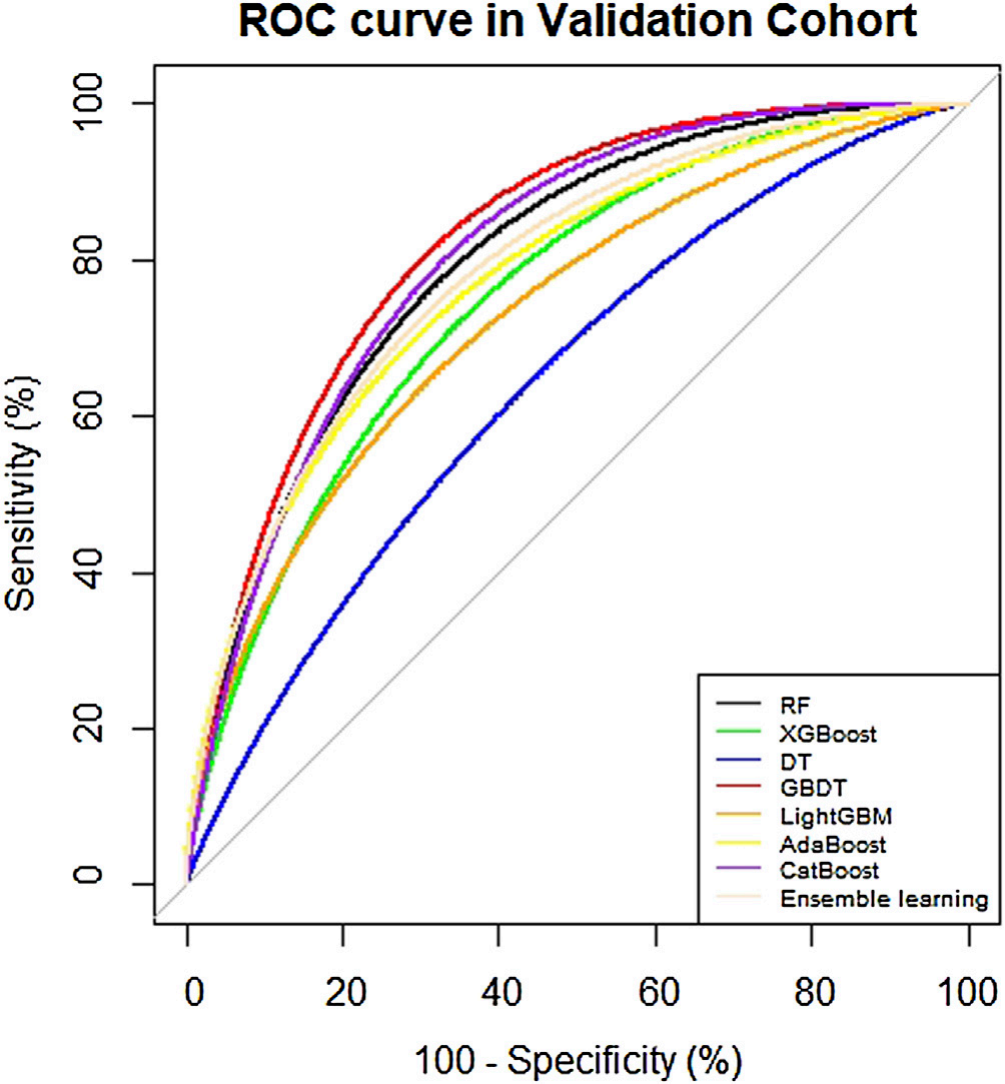
ROC curve of eight models in the test cohort.

**FIGURE 6:**
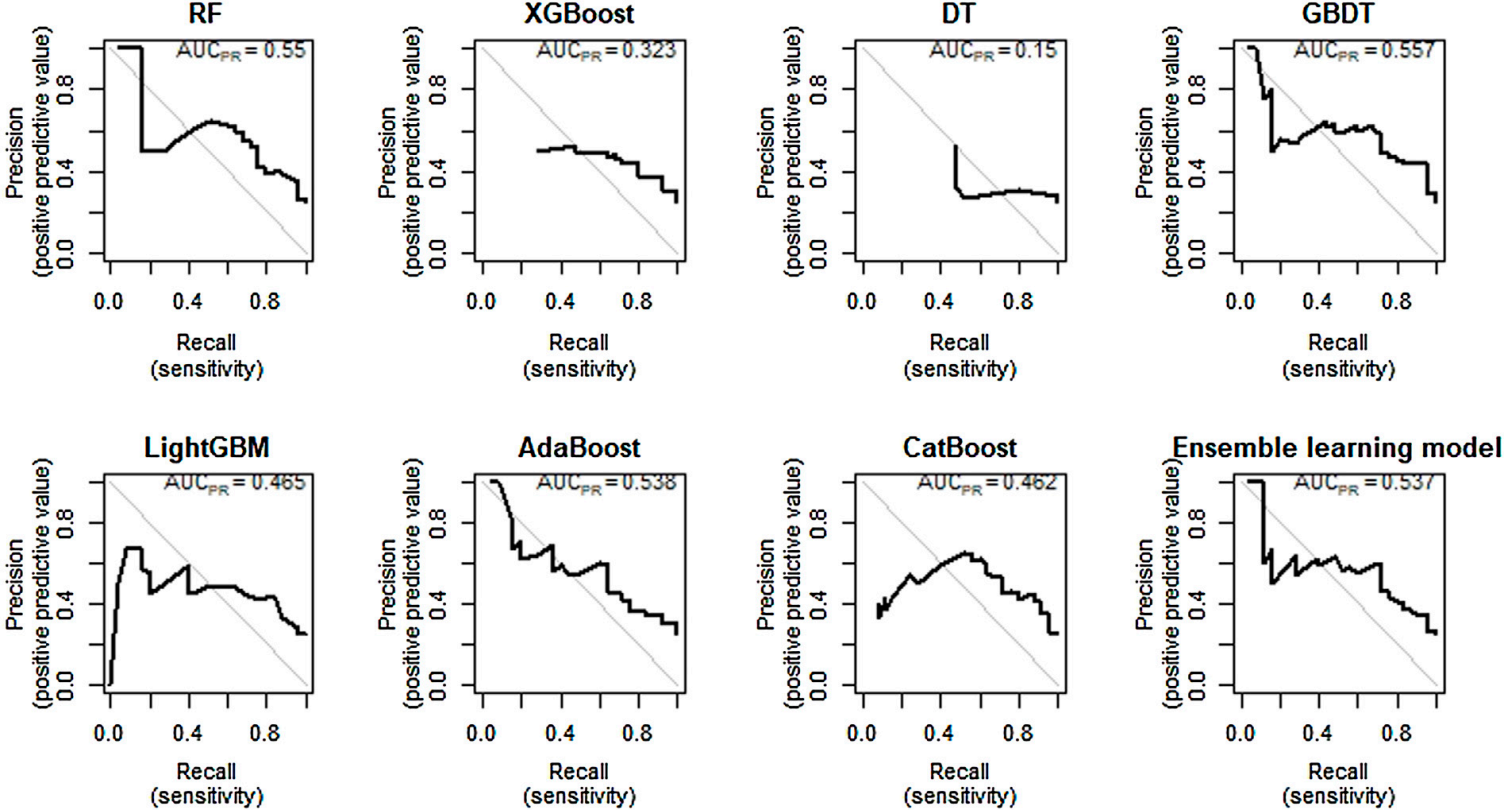
PR curve of eight models in the test cohort.

**FIGURE 7:**
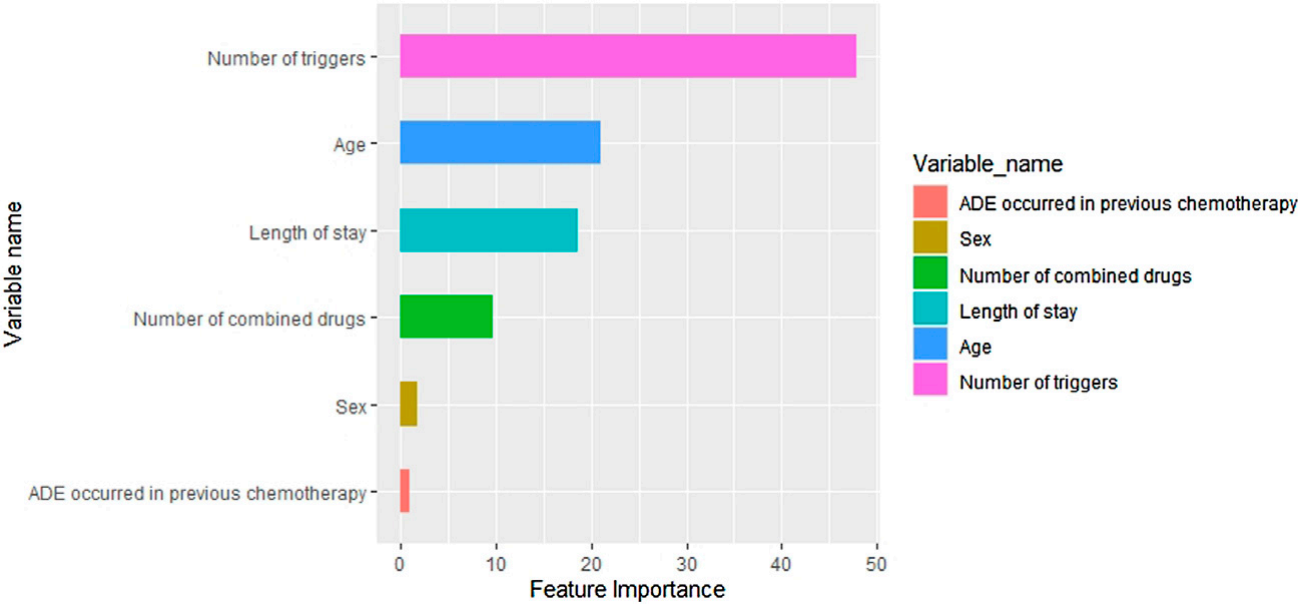
Ranking of variable importance in the GBDT model.

In Table 5, the metrics of eight models were compared in terms of SEN, SPE, AUROC, AUPR etc. in the test cohort. Among the eight models, the GBDT has the highest precision (0.621) and with the highest values of F1 (0.667), but owns a moderate recall (0.720). In addition, the visual comparisons of the ROC are shown in Figure 5, where the GBDT model achieves the highest AUROC of 0.832 and higher than the nomogram’s AUROC of 0.799. The SPE of the GBDT model was 0.853, suggesting that the GBDT model also has good value in identifying SAE-negative patients. Figure 6 shows the PR curves of the eight models, the GBDT model also outperforms the other seven models, with the AUPR of 0.557. It can be seen that the GBDT model outperforms the other models in the aspect of precision, F1, AUPR, and AUROC, demonstrating a good ability for model prediction. Under overall consideration of the predicting performance, we chose the model using the GBDT algorithm over the others to predict the occurrence of SAEs. Among the GBDT model, the importance of six variables ranks as follows: number of triggers, age, number of combined drugs, length of stay, ADEs occurred in previous chemotherapy, and sex (Figure 7). In addition, our webpage SAE risk prediction calculator using the GBDT algorithm model can be accessed through https://cqmugj.shinyapps.io/SAEs_diagnostic__tools/.

**TABLE 5:** Eight algorithms’ model performance in the test cohort.

Model	AUROC	SEN	SPE	AUPR	Precision	Recall	F1
RF	0.805 (0.705, 0.906)	0.760	0.773	0.550	0.528	0.760	0.623
XGBoost	0.754 (0.645, 0.863)	0.760	0.720	0.323	0.475	0.760	0.585
DT	0.650 (0.525, 0.776)	0.480	0.853	0.150	0.522	0.480	0.500
GBDT	0.832 (0.744, 0.920)	0.720	0.853	0.557	0.621	0.720	0.667
LightGBM	0.750 (0.635, 0.864)	0.840	0.640	0.485	0.438	0.840	0.575
AdaBoost	0.782 (0.678, 0.886)	0.640	0.867	0.538	0.615	0.640	0.627
CatBoost	0.817 (0.725, 0.909)	0.720	0.813	0.462	0.563	0.720	0.632
Ensemble learning model	0.797 (0.694, 0.899)	0.720	0.840	0.537	0.600	0.720	0.655

## 4 Discussion

Medical electronic records have developed from data storage to data utilization, which can potentially guide clinical decision-making and predict important results (Ibrahim et al., 2020). It is a low-cost, feasible, and effective method to use medical electronic records and machine learning algorithms to predict the occurrence of SAEs of antineoplastic drugs. We first made a preliminary analysis of ADEs of antineoplastic drugs by the GTT method that used the data of 499 cancer inpatients, and SAEs were identified from patients with ADEs. After that, we constructed a probability prediction model of SAEs in cancer inpatients using the nomogram and machine learning method so that clinical workers can intervene in time when SAEs occurred.

We observed that the risk factors of SAEs in cancer inpatients were the number of triggers, length of stay, age, number of previous chemotherapies, ADEs occurred in previous chemotherapy, and sex. Similar to the study of Ze et al. (2021), our study also introduced the number of triggers as a variable in the prediction model. We found that the number of triggers is the most important risk factor. Increasing the number of triggers could better predict the probability of SAEs of antineoplastic drugs. The GTT studies are characterized by a great methodological heterogeneity because the GTT is typically adapted to the local context by removing modules (Härkänen, 2014; Doupi et al., 2015; Hibbert et al., 2016; Xiao-Di et al., 2016; Jee-In et al., 2018), adding triggers and specific definitions (Lau and Kirkwood, 2014), or adding new modules before implementation. A German study which focuses on ADE identification in surgery and neurosurgery shows that new triggers should be added in the process of identifying ADEs to adapt to the new environment (Mareen et al., 2019). Therefore, we suggest that the number of triggers should be combined with other important risk factors to predict SAEs better.

We also confirmed three risk factors which were the length of stay, age, and sex. These three risk factors were proved in previous studies (Nazer et al., 2014; Christin et al., 2017; Weingart et al., 2020). Previous researchers have proved that there is a strong correlation between the length of stay and the incidence of ADEs (Classen et al., 2011; Sezgin et al., 2013). The risk of ADEs increases by 5.1% every day (Christin et al., 2017). However, the length of stay is usually affected by other factors, such as the severity of the disease. Moreover, the increase in the length of stay may be a result of the occurrence of SAEs. Therefore, the causal relationship between the length of stay and SAEs needs to be further evaluated. In addition, age is also an important risk factor. This may be related to more types of drugs used in younger patients. In our study, the number of previous chemotherapies and number of combined drugs in younger patients were higher than those in older patients (Andrew and Lisa, 2012). It should be noted that in the field of drug treatment and drug delivery, some investigators have discovered that sex differences could influence pharmacokinetics and pharmacodynamics and drug toxicity (Bernd et al., 2002; Janice, 2003). However, in this study, sex is a risk factor for SAEs in cancer inpatients which is inconclusive in existing studies. Therefore, further research is required on this factor.

Of note, we also found that number of previous chemotherapies and any ADEs in previous were also risk factors for SAEs in cancer inpatients. The potential reason for the positive correlation between SAEs and the number of previous chemotherapies and was there any ADEs in previous may be the two factors leading to the worse physical state of patients, and SAEs are more likely to occur in the case of poor physical state (Ekkamol et al., 2018).

From the perspective of the overall performance of the model, the performance of the logistic-based nomogram was not as good as the performance based on the machine learning algorithm.

Logistic regression is widely used in the medical field to explore the risk factors of diseases because of its strong interpretability. The transparency of the nomogram established based on the logistic model could solve the black box problem of the machine learning model, but it has the disadvantage of underfitting when building the model, and the overall performance of the model is not high. However, the indicators selected by machine learning were more than those selected by the nomogram in this study, which may be one of the reasons why the performance of machine learning was better than the nomogram.

Machine learning is an emerging artificial intelligence discipline that can describe the complex non-linear relationship between independent variables and dependent variables, and the resulting impressive forecast ability (Fabrizio et al., 2021). In our study, the AUROC values of the algorithms other than the DT algorithm reached more than 0.7, indicating good predictive ability. The DT is a traditional machine learning algorithm that can build a classification model based on the information gained from the predictors, so it is optimal in terms of model interpretability (Höppner, 2020). However, the decision tree algorithm is easy to fall into overfitting, and it is easy to fall into local optimum. It has been proved in many works of literature that its performance is not as good as other algorithms. Compared with other machine learning models, the GBDT has the best comprehensive performance, with an AUROC of 0.832 (0.744, 0.920), and an AUPR of 0.557. The possible reason is that the six predictors were finally included in this study, and the GBDT algorithm has obvious advantages over the other machine learning algorithms in dealing with low-dimensional and non-linear data (Yuhui et al., 2022). In addition, light GBM has the highest SEN (0.840) and AdaBoost has the highest SPE (0.863), suggesting that they have an advantage in predicting positive and negative cases. Furthermore, we also built an ensemble learning model combining the results of the seven algorithms, with an AUROC of 0.797 (0.694, 0.899), and an AUPR of 0.557. Ensemble learning achieves significantly better generalization performance than a single learner by combining multiple learners and also achieves good results in our dataset (Makoto et al., 2021; Menglin et al., 2021).

In this study, we established a prediction model for SAEs of cancer inpatients using antineoplastic drugs. Researchers can incorporate the risk factors identified in our study into web pages to determine the probability of SAE occurrence in cancer inpatients. However, this study also has some limitations. This study was a retrospective study and may lack some valuable features that limit the selection of variables for modeling. Furthermore, this study was a single center and small sample study, which fails to externally verify the prediction results of the model in multi-center and large samples. In the future, a large-scale, multi-center, and prospective study is needed for verification.

## Data Availability

The original contributions presented in the study are included in the article/Supplementary Material; further inquiries can be directed to the corresponding authors.
